# Prognosis of pancreatic cancer with Trousseau syndrome: a systematic review of case reports in Japanese literature

**DOI:** 10.1186/s43046-023-00202-2

**Published:** 2023-12-14

**Authors:** Munehiro Wakabayashi, Yoshinori Kikuchi, Kazuhisa Yamaguchi, Takahisa Matsuda

**Affiliations:** 1 Division of Gastroenterology and Hepatology, Department of Internal Medicine (Omori), Faculty of Medicine, School of Medicine, Toho University, Tokyo, Japan; 2 Department of Clinical Oncology, Faculty of Medicine, Toho University, Tokyo, 143-8541 Japan

**Keywords:** Pancreatic cancer, Trousseau syndrome, Prognosis, Chemotherapy

## Abstract

Trousseau syndrome is a paraneoplastic syndrome associated with a risk of poor prognosis. We reviewed the survival time and prognosis of patients with Trousseau syndrome. We identified 40 cases from 28 reports of Trousseau syndrome due to pancreatic cancer. We analyzed 20 cases based on reports providing sufficient information on the stage/location of pancreatic cancer and survival time after Trousseau syndrome. The median survival time was 2.0 months. There was no statistical difference between performance status (PS) 0–1 and PS 4, stages I–III and IV, and pancreatic head and body/tail. However, statistically significant differences were noted between the median survival time of patients who continued treatment for pancreatic cancer even after Trousseau syndrome and those who discontinued treatment (*P* = 0.005). Although only a small number of cases were analyzed in this study, the results indicated that patients with pancreatic cancer who developed Trousseau syndrome had a poor prognosis, and chemotherapy should be continued, if possible.

## Background

Trousseau syndrome is a hypercoagulable condition or disseminated intravascular coagulation (DIC) associated with malignancy and migratory thrombophlebitis [1]. However, in Japan, Trousseau syndrome is often narrowly defined as cerebral infarction associated with malignancy. In autopsy cases of patients with cancer, nonbacterial thrombotic endocarditis (NBTE) is the most common cause of stroke (18.5%), followed by DIC (9.6%) [2]. Furthermore, 51.6% of patients with NBTE had malignancy, whereas 41.9% had DIC, indicating that NBTE is a mechanism of embolism in large cerebral cortical branches [3]. Therefore, Trousseau syndrome is considered a systemic arterial embolism caused by venous thromboembolism (VTE) or NBTE based on hypercoagulable conditions, such as DIC associated with malignant tumors [4].

Thromboembolism is the second leading cause of death in patients with malignant tumors [5]. The median survival time (MST) after the development of Trousseau syndrome is 4.5 months, which exhibits a poor prognosis [6].

According to cancer-related statistical data in Japan, the 5-year relative survival rate of patients with pancreatic cancer is 8.5%, and it showed the fourth highest mortality rate in 2020, making it a malignant disease with a poor prognosis whose incidence has been increasing in recent years [7]. This indicates that pancreatic cancer associated with Trousseau syndrome has a poor prognosis; however, no such reports have been published so far. Therefore, we investigated the prognosis of patients with pancreatic cancer and Trousseau syndrome.

## Methods

Using the Ichushi-Web database, case reports published between January 1964 and March 2022 were screened using the keywords “pancreatic cancer” and “Trousseau syndrome.” Among 28 Japanese case reports, we identified 40 patients with pancreatic cancer and Trousseau syndrome [8–35]. Furthermore, we excluded patients with unknown survival time and treatment status after the onset of Trousseau syndrome [10, 13, 16, 20, 22, 26, 27, 33]. Finally, 20 patients were included in this study. The Eastern Cooperative Oncology Group performance status (PS) was based on the status at the onset of Trousseau syndrome.

### Statistical analysis

Kaplan–Meier survival curves were obtained using the survival time from pancreatic cancer or Trousseau syndrome diagnosis, as described in previous reports. The log-rank test was used to assess the differences in survival time. Hazard ratios were calculated using a Cox proportional hazards model. Fisher’s exact test was used to compare groups with and without chemotherapy after the onset of Trousseau syndrome. All statistical analyses were performed using EZR Ver.1.55 (Saitama Medical Center, Jichi Medical University, Saitama, Japan) [36]. *p*-values of < 0.05 were considered to indicate statistical significance.

## Results

### Patient characteristics

The clinical features of 20 patients with pancreatic cancer and Trousseau syndrome are listed in Table 1. There were 8 male and 12 female patients. The number of patients by age group was as follows: one in their 50 s, five in their 60 s, six in their 70 s, seven in their 80 s, and one in their 90 s. The PS was 0–1 in 7 cases and 4 in 13 cases. The primary site of pancreatic cancer was the pancreatic head in 9 cases and pancreatic body/tail in 11 cases. The clinical staging of patients was as follows: stages I–III in 5 cases and stage IV in 15 cases. The treatment classification after Trousseau syndrome was “nontreatment” in 14 cases, “gemcitabine alone” in 3 cases, “gemcitabine plus nab-paclitaxel” in 1 case, and “tegafur–gimeracil–oteracil potassium” in 2 cases.

**Table 1:** Baseline characteristics of the patients

**Variables**	**Number(*n*=20)**	
Gender	Male	8
	Female	12
Age	50-59	1
	60-69	5
	70-79	6
	80-89	7
	>90	1
Performance Status	0-1	7
	4	13
Primary site	Head	9
	body/tail	11
Stage	I	1
	II	1
	III	3
	IV	15
Treatment after the onset of Trousseau syndrome		
	None	14
	Gemcitabine	3
	Gemcitabine plus nab-Paclitaxel	1
	Tegafur-gimeracil-oteracil potassium	2

### Symptoms of Trousseau syndrome

**Fig. 1:**
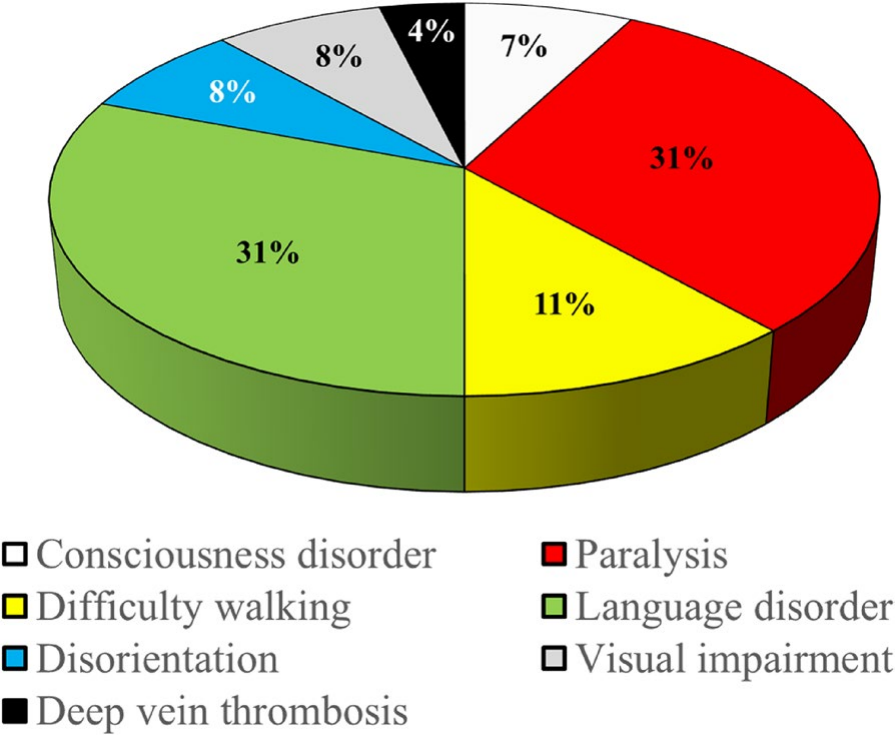
Symptom frequency of Trousseau syndrome

Symptoms of all 20 patients at the onset of Trousseau syndrome were compiled. All overlapping symptoms were identified: 7% of patients had consciousness disorder, 31% had paralysis, 11% had difficulty walking, 31% had language disorders, 8% had disorientation, 8% had visual impairment, and 4% had deep vein thrombosis (Fig. 1). The most frequent movement disorders were paralysis, difficulty walking, and speech disorders.

### Survival time for each factor

**Fig. 2:**
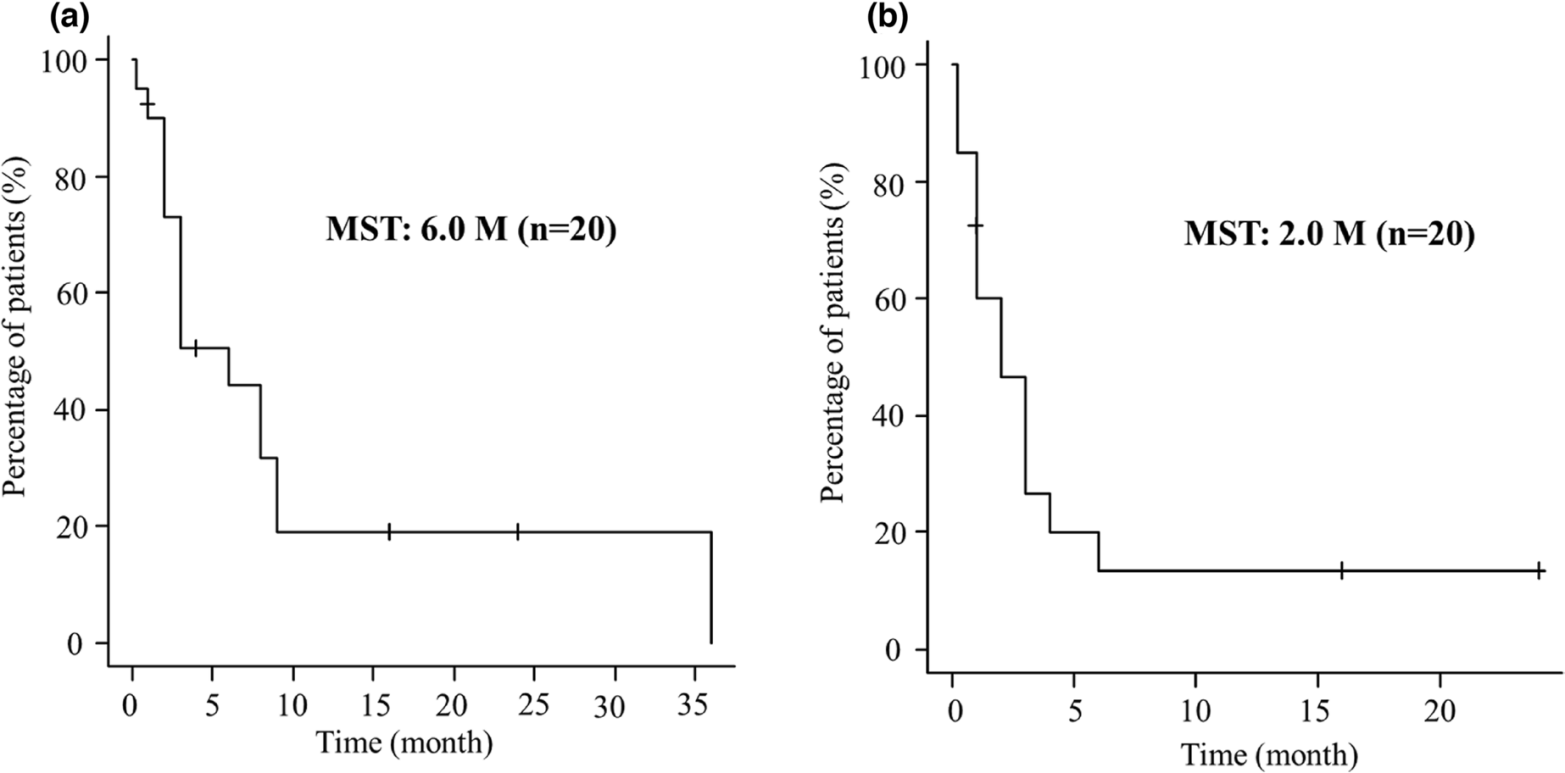
Overall survival curve of all 20 cases. **a** Overall survival after diagnosis of pancreatic cancer. **b** Overall survival from the onset of Trousseau syndrome

**Fig. 3:**
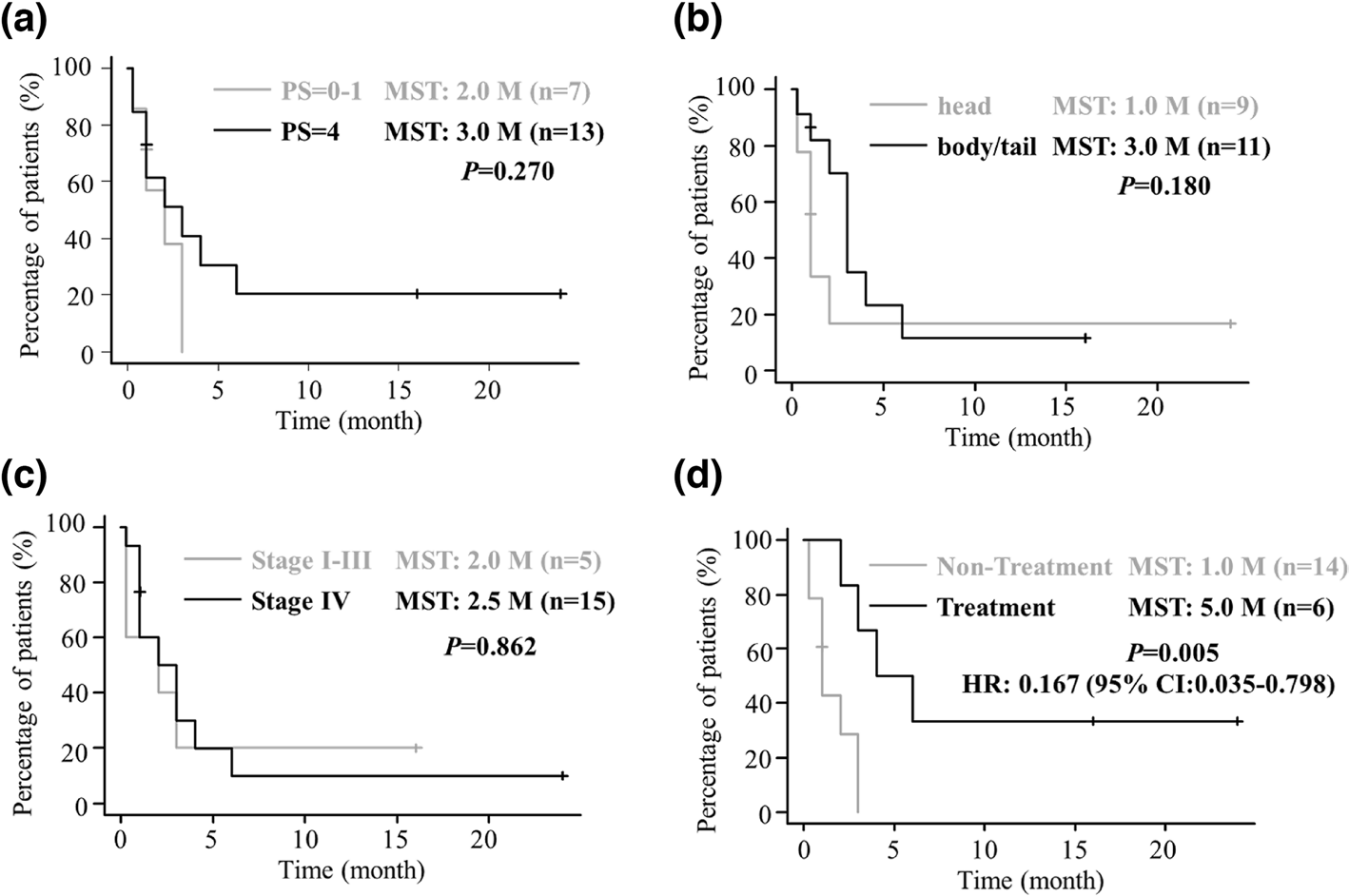
Overall survival curves for each factor (PS, primary site, stage, and continuation of treatment for pancreatic cancer). **a** Overall survival curves in PS 0–1 and 4. **b** Overall survival curves in the pancreatic head/uncinate process and pancreatic body/tail. **c** Overall survival curves in stages I–III and IV. **d** Overall survival curves in groups with and without pancreatic cancer treatment after the onset of Trousseau syndrome

The MST for all 20 reported cases was 6.0 months from pancreatic cancer diagnosis and 2.0 months from Trousseau syndrome onset (Fig. 2). Upon comparing each factor in pancreatic cancer and Trousseau syndrome, the MST for PS 0–1 (*n* = 7) was 2.0 months, and that for PS 4 (*n* = 13) was 3.0 months, with no significant difference between the two groups (*P* = 0.270; Fig. 3a). The MST for the pancreatic head (*n* = 9) was 1 month, and that for the pancreatic body/tail (*n* = 11) was 3.0 months, with no significant difference between the two groups (*P* = 0.180; Fig. 3b). The MST for stages I–III (*n* = 5) was 2.0 months, and that for stage IV (*n* = 15) was 2.5 months, with no significant difference between the two groups (*P* = 0.862; Fig. 3c). However, the MST with and without chemotherapy after Trousseau syndrome was 1.0 month in the no treatment (*n* = 14) group and 5.0 months in the treatment (*n* = 6) group, with a significantly longer survival time in the treatment group (*P* = 0.005; Fig. 3d).

### Frequency of chemotherapy administration after Trousseau syndrome diagnosis according to PS, primary site, and stage

**Fig. 4:**
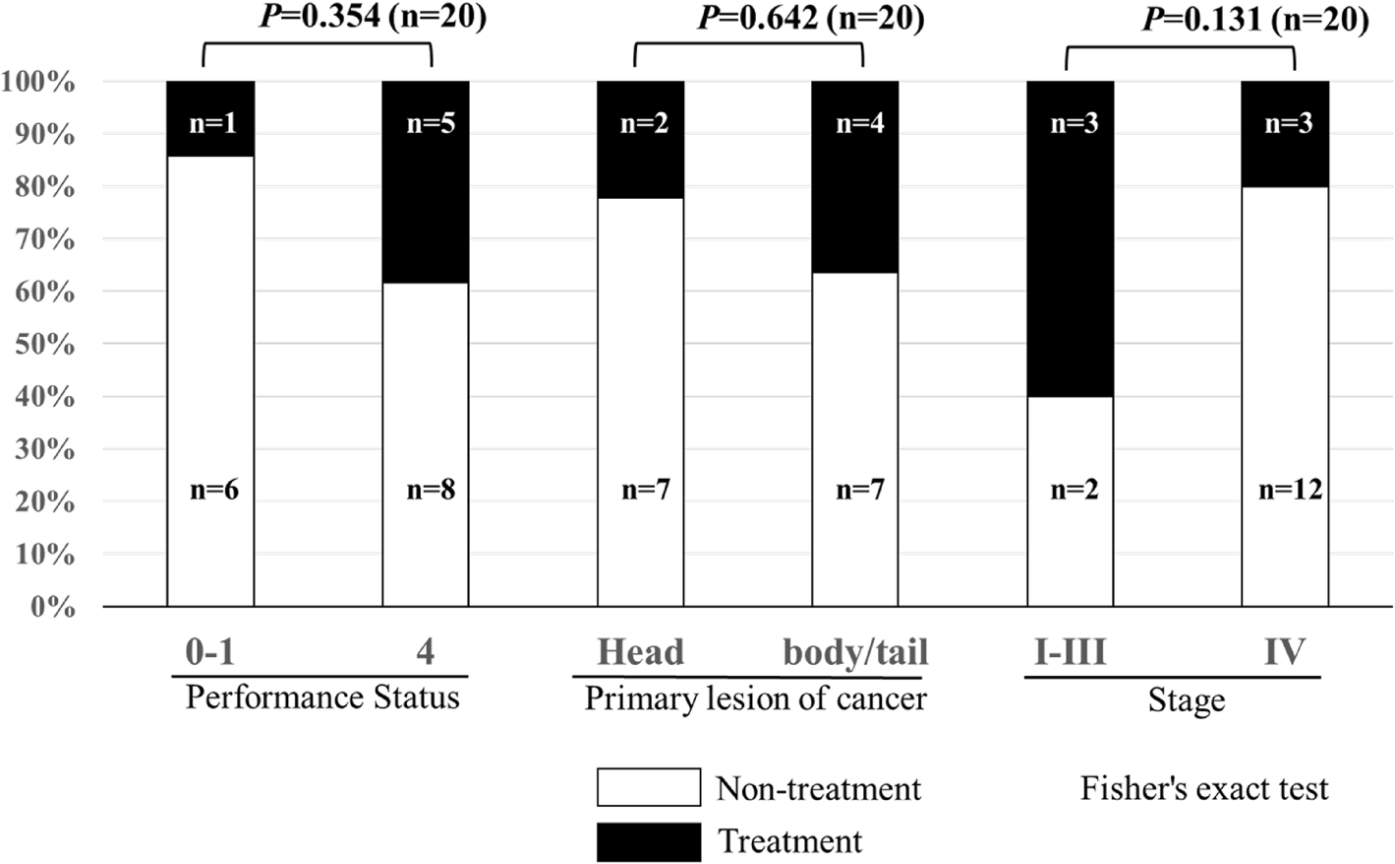
Frequency of each factor with and without chemotherapy. **a** Performance status 0–1 and 4. **b** Pancreatic head and body/tail. **c** Stages I–III and IV

We compared the frequency of chemotherapy administration after Trousseau syndrome diagnosis according to PS, primary site, and stage. No significant difference was noted between PS 0–1 (no treatment, *n* = 6 and treatment, *n* = 1) and PS 4 (no treatment: *n* = 8 and treatment: *n* = 5) (*P* = 0.354; Fig. 4a). Furthermore, no significant difference was observed between the pancreatic head (no treatment, *n* = 7 and treatment, *n* = 2) and pancreatic body/tail (no treatment, *n* = 7 and treatment, *n* = 4) (*P* = 0.642; Fig. 4b). There was no significant difference between stages I–III (no treatment, *n* = 2 and treatment, *n* = 3) and stage IV (no treatment, *n* = 12 and treatment, *n* = 3) (*P* = 0.131; Fig. 4c).

## Discussion

This study revealed that patients with pancreatic cancer who developed Trousseau syndrome had a poor prognosis, with an MST of 2.0 months. However, patients who continued to receive chemotherapy after developing Trousseau syndrome had a significantly longer prognosis than those who did not. In general, PS4 or stages I–III pancreatic cancer is not an indication for chemotherapy; however, this study suggests that chemotherapy prolongs survival in patients with pancreatic cancer complicated by Trousseau syndrome.

In 1865, Armand Trousseau reported that thrombophlebitis and venous thrombus were efficiently complicated in patients [37]. Malignant tumors and thromboembolisms are closely related, and the concept of cancer-associated thromboembolism (CAT), which includes deep vein thrombosis and pulmonary thromboembolism, has been previously proposed. Trousseau syndrome is considered a case of CAT [38].

Depending on the location, stroke may cause consciousness disorder, delirium, paralysis, sensory disturbance, convulsions, visual disturbance, and cerebellar damage [4]. Paralysis and language disorders were the most common symptoms. Most patients with cancer are asymptomatic; therefore, it is crucial to screen for malignant diseases when stroke occurs. In particular, cancers associated with a high VTE risk in outpatients may affect the stomach and pancreas [39].

Owing to advances in treatment methods, the MST for pancreatic cancer in Japan tended to increase from 5.2 (1981–1990) and 6.5 (1991–2000) to 10.2 months (2001–2004). From 2001 to 2004, the MST for resectable (stages I–III) and unresectable (stage IV) cases was reported to be 18.2 and 7.8 months, respectively [40]. This study revealed that the MST after pancreatic cancer diagnosis is very short (6.0 months), and that after Trousseau syndrome, onset is extremely short (2.0 months). Similarly, the MST after stroke in patients with cancer at the Memorial Sloan–Kettering Cancer Center was reported to be 4.5 months, and 25% of patients died within 30 days [5]. Hence, Trousseau syndrome may be a poor prognostic factor for cancer.

The PS of > 1 has been reported to be a poor prognostic factor for pancreatic cancer throughout all stages of the disease [41, 42]. Stage IV has a poorer prognosis than stages I–III [39]. Furthermore, the incidence of thrombosis has been reported to be higher in carcinomas of the pancreatic body/tail than in those of the pancreatic head [43]. We previously reported that pancreatic body/tail cancer has a poorer prognosis than pancreatic head cancer, and that chemotherapy as a second-line treatment can improve prognosis [44]. Similarly, a study comparing chemotherapy and best supportive care as second-line treatments for unresectable pancreatic cancer reported a significant survival benefit in the chemotherapy group [45]. Therefore, we compared survival according to four factors: PS, primary site, staging, and chemotherapy. The results showed no significant differences in survival according to PS, primary site, and staging. Alternatively, those who received chemotherapy after developing Trousseau syndrome showed significantly longer survival than those who discontinued chemotherapy.

Koprowski et al. reported carbohydrate antigen 19–9 (CA19-9) as a gastrointestinal cancer-related antigen [46], which is present in the blood as a mucin-type glycoprotein and has a sialyl-Lewis A sugar chain as an antigenic determinant. High serum CA19-9 levels have been reported in pancreatic, biliary tract, colorectal, and liver cancers, with an exceptionally high incidence in pancreatic and biliary tract cancers [47]. Furthermore, the highest microparticle-associated tissue factor activity and CA19-9 levels have been reported in patients with pancreatic cancer who developed thrombosis or cerebral embolism despite anticoagulation therapy [48]. Further, a previous study revealed that Trousseau syndrome is most likely caused by the interaction between circulating cancer mucins and leukocyte L-selectin and platelet P-selectin, without thrombin generation [49]. Similar to CA19-9, Trousseau syndrome has been reported to be common in adenocarcinomas, especially mucin-producing tumors such as lung, pancreatic, gastric, ovarian, and breast cancers [50, 51]. These findings suggest that pancreatic cancer, CA19-9, and Trousseau syndrome are closely related.

However, chemotherapy has been reported to be a causative factor of thrombosis [52–55]. The mechanisms of thrombosis during chemotherapy include the release of procoagulant substances and cytokines from tumor cells due to cell-targeted therapy, damage to the vascular endothelium owing to chemotherapy, and decrease in the levels of natural anticoagulants (protein C, protein S, and antithrombin) [55]. Therefore, chemotherapy may aggravate Trousseau syndrome.

Varki suggested that the primary approach for treating Trousseau syndrome is to remove the causative tumor [56]. However, tumor removal is not always possible. In such cases, it is preferable to reduce the tumor volume using chemotherapy. No clinical studies have shown an improvement in patients with Trousseau syndrome using chemotherapy, but a case report of long-term survival with a combination of chemotherapy and heparin therapy has been reported [57]. Regarding DIC syndrome in patients with cancer, a combination of recombinant human soluble thrombomodulin with chemotherapy has been reported to significantly prolong survival in the no-chemotherapy group [58]. This report also supports our findings.

The current study is a systematic review of a few cases; therefore, multivariate analysis is impossible. Furthermore, as certain case reports had excluded the effect of chemotherapy on tumors or CA19-9 values, the results could not be thoroughly investigated. Therefore, further investigation is warranted to determine whether chemotherapy improves the prognosis of patients with Trousseau syndrome. In this study, we also accumulated case reports of Trousseau syndrome in patients with pancreatic cancer in Japan. However, the possibility of cerebral infarction due to causes other than cancer (cardiac disease and aging) cannot be excluded; it is extremely challenging to distinguish Trousseau syndrome from normal cerebral infarction. Thus, it is necessary to clarify the diagnostic criteria for cerebral infarction due to Trousseau syndrome in the future.

## Conclusion

This study suggests that combining heparin therapy and chemotherapy contributes to survival in patients with pancreatic cancer complicated by Trousseau syndrome if their general condition is acceptable, even in the presence of paralysis.

## Data Availability

The datasets generated and/or analyzed during the current study are available from the corresponding author upon reasonable request. Derived data supporting the findings of this study are available from the corresponding author Y. K. on request.

## Abbreviations

CAT: Cancer-associated thromboembolism
DIC: Disseminated intravascular coagulation
MST: Median survival time
PS: Performance status
